# Impact of Patient Profile on CDK4/6 Inhibitor Therapy Outcomes: A Real-World Data Analysis

**DOI:** 10.3390/ijms26073357

**Published:** 2025-04-03

**Authors:** Ioana-Miruna Stanciu, Maria-Cristina Orlov-Slavu, Andreea-Ioana Parosanu, Cornelia Nitipir

**Affiliations:** 1 Department of Oncology, “Carol Davila” University of Medicine and Pharmacy, 020021 Bucharest, Romania; 2 Emergency Hospital, Elias University, 011461 Bucharest, Romania; 3 “Prof. Dr. Agrippa Ionescu” Emergency Clinical Hospital, 707961 Balotesti, Romania

**Keywords:** CDK4/6 inhibitors, real-world data, patient profile, HR+/HER2− metastatic breast cancer, outcome, PFS, OS

## Abstract

Cyclin-dependent kinase 4 and 6 (CDK4/6) inhibitors have transformed the treatment landscape for patients with hormone receptor-positive (HR+)/HER2-negative (HER2−) breast cancer. However, their efficacy is influenced by various clinical and biological factors, including patient age, tumor biology, and treatment-related toxicities. The aim of this study is to evaluate the impact of demographic, clinical, and tumor-related characteristics on the efficacy of CDK4/6 inhibitors in a cohort of patients with metastatic HR+/HER2− breast cancer. We conducted a retrospective cohort study analyzing the outcomes of 95 patients with metastatic ER-positive, HER2-negative breast cancer (BC) treated with CDK4/6 inhibitors (ribociclib, palbociclib, and abemaciclib) in combination with endocrine therapy. The patient demographics, tumor characteristics, and treatment regimens were examined, with a primary focus on progression-free survival (PFS), overall survival (OS), time on treatment (TOT), and the influence of clinical and biological factors. Younger patients (under 50 years) demonstrated higher tumor aggressiveness, reflected by higher Ki67 levels and histological grades, which negatively impacted their survival outcomes. Ribociclib was associated with the highest survival benefit, particularly in younger patients. Older patients (over 50 years) showed greater rates of comorbidities and toxicity, with dose reductions correlated with improved survival outcomes. This study highlights the significance of personalized treatment strategies based on patient age, comorbidities, and tumor biology. Ribociclib shows superior efficacy in younger, less comorbid patients, while palbociclib remains a viable option for older patients with higher comorbidity burdens.

## 1. Introduction

Breast cancer (BC) remains one of the most common and deadly cancers worldwide, with estrogen receptor (ER) positivity being a hallmark of approximately 73% of breast cancer cases [1]. In patients with metastatic ER-positive and human epidermal growth factor receptor 2 (HER2)-negative breast cancer, survival rates remain a major concern, with the 5-year relative survival rate for these patients reported to be as low as 34% [2]. The management of metastatic ER-positive HER2-negative breast cancer has evolved with the development of targeted therapies aimed at prolonging progression-free survival (PFS) and overall survival (OS).

Cyclin-dependent kinase 4/6 inhibitors (CDK4/6i) represent a major advancement in the treatment of this subtype of breast cancer. These agents, which include palbociclib, ribociclib, and abemaciclib, inhibit CDK 4 and CDK 6, proteins that are involved in cell cycle regulation and tumor progression in ER-positive breast cancer. The use of CDK4/6 inhibitors in combination with endocrine therapy (ET) is now considered the standard of care for patients with advanced or metastatic ER-positive, HER2-negative breast cancer [3,4,5]. Recent clinical trials and real-world evidence have demonstrated that the addition of CDK4/6 inhibitors to endocrine therapy significantly improves PFS, with the median PFS in various studies ranging from 18 to 27 months depending on the specific inhibitor and patient characteristics [6,7,8,9].

For example, the PALOMA-2 trial, which evaluated palbociclib in combination with letrozole, reported a median PFS of 24.8 months, while the MONALEESA-2 trial demonstrated a median PFS of 25.3 months with ribociclib and letrozole [10,11]. These findings were further corroborated by the MONARCH-3 trial with abemaciclib, which showed a median PFS of 28 months when combined with letrozole [12]. The clinical efficacy of CDK4/6 inhibitors has transformed the landscape of metastatic breast cancer treatment, with improved outcomes even in the presence of certain mutations that contribute to endocrine resistance [5,13].

However, real-world data on the outcomes of CDK4/6 inhibitors outside the controlled setting of clinical trials remain sparse. While pivotal clinical trials have established the efficacy and safety of CDK4/6 inhibitors in HR+/HER2− metastatic breast cancer, real-world data remain limited. Specifically, there is a lack of comprehensive evidence on their treatment patterns in routine clinical practice and the influence of patient-specific factors, such as comorbidities, prior therapies, and the presence of metastases at diagnoses. This study aims to bridge these gaps by analyzing real-world outcomes in a diverse patient population, providing insights beyond the controlled environment of clinical trials.

Studies have highlighted that factors such as patient age, comorbidities, and prior lines of therapy may influence treatment outcomes [14,15,16]. In addition, a growing body of evidence has pointed to the role of genetic alterations, such as mutations in PIK3CA or loss of PTEN, which may predict a differential response to CDK4/6i therapy [17,18]. Recent cohort studies have found that the overall survival (OS) for patients receiving first-line CDK4/6 inhibitors can range from 30 to 50 months, with time on treatment averaging from 14 to 16 months [19,20,21].

In this context, real-world data become crucial to evaluate the effectiveness of CDK4/6 inhibitors in routine clinical practice and to identify patient characteristics that impact treatment outcomes. Despite the positive outcomes observed in clinical trials, variability in patient responses suggests the importance of personalized medicine strategies. In particular, patient profiles, such as age, ECOG performance status, prior treatments, and comorbid conditions, can significantly impact PFS and OS [22,23]. A study [24] analyzing 274 metastatic breast cancer patients treated with CDK4/6 inhibitors found that those with an Eastern Cooperative Oncology Group (ECOG) performance status of 0 had a median PFS of 23.4 months if younger than 70 years and 27.2 months if 70 years or older. In contrast, patients with an ECOG score of 1 experienced shorter PFS, highlighting the impact of performance status on treatment outcomes. The presence of comorbidities also affects survival outcomes. A multivariable analysis [19] indicated that patients with visceral metastases had a higher risk of disease progression (HR, 1.45; 95% CI, 1.17–1.80; *p* = 0.008) and mortality (HR, 1.63; 95% CI, 1.19–2.24; *p* = 0.002), underscoring the importance of considering comorbid conditions in treatment planning.

To better understand the impact of CDK4/6 inhibitors in a real-world setting, we conducted a retrospective study at a cancer center in Romania. Our study aimed to evaluate the clinical outcomes associated with all three CDK4/6 inhibitors used in combination with endocrine therapy. This is the first study in Romania to explore the effects of CDK4/6 inhibitors in a real-world patient cohort, with a focus on how specific patient characteristics influence treatment outcomes. Our findings contribute to the growing body of evidence on the effectiveness of CDK4/6 inhibitors and highlight the importance of individualized treatment approaches in metastatic ER-positive HER2-negative breast cancer.

## 2. Results

### 2.1. Clinical and Biological Characteristics of Patients

Patient demographic and clinical characteristics (Table 1) assessed at baseline included age at diagnosis, area of residence, histological grade, stage at diagnosis (locally advanced vs. metastatic), metastasis localization, bone-only metastatic disease, number of disease sites, type of CDK4/6 inhibitor used, duration of CDK4/6 inhibitor therapy, associated endocrine therapy, menopausal status, Ki67 percentage, CA15-5 levels at diagnosis, comorbidities, survival status, family cancer history, prior chemotherapy (before or after CDK4/6 inhibitor therapy), mastectomy or radiotherapy before CDK4/6i treatment, and NLR (neutrophile-to-lymphocyte) ratio.

Premenopausal and perimenopausal patients included in this study received goserelin for ovarian function suppression as part of their treatment regimen.

The study population had a mean age of 57.08 ± 13.03 years. Of the 95 patients, 51.6% were under 50 years of age, and 48.4% were over 50 years. Most patients (82.1%) were postmenopausal. The majority presented with stage II (66.3%) and stage III (24.2%) disease at diagnosis, while 41.1% had metastatic disease. Bone-only metastasis was seen in 44.2% of the patients, while visceral metastases were present in 40%.

Regarding tumor grade, 66.3% of the patients had G2 tumors, while G3 tumors were more common in the younger patients (23.3%) compared to the older patients (18.6%). Ki67 expression was elevated (>20%) in 54.7% of the patients, with higher levels observed in the younger patients, particularly those with G3 tumors.

### 2.2. Treatment Characteristics

The most frequently prescribed CDK4/6 inhibitor was palbociclib (43.2%), followed by ribociclib (39.0%) and abemaciclib (17.8%). The younger patients received ribociclib more often (34.8%), whereas the older patients were more frequently treated with palbociclib (64.5%).

The median treatment duration was 22.63 months for palbociclib, 20.56 months for ribociclib, and 11.67 months for abemaciclib. Notably, the patients receiving abemaciclib had the shortest treatment duration, which may reflect earlier disease progression or intolerance to the treatment.

**Figure 1:**
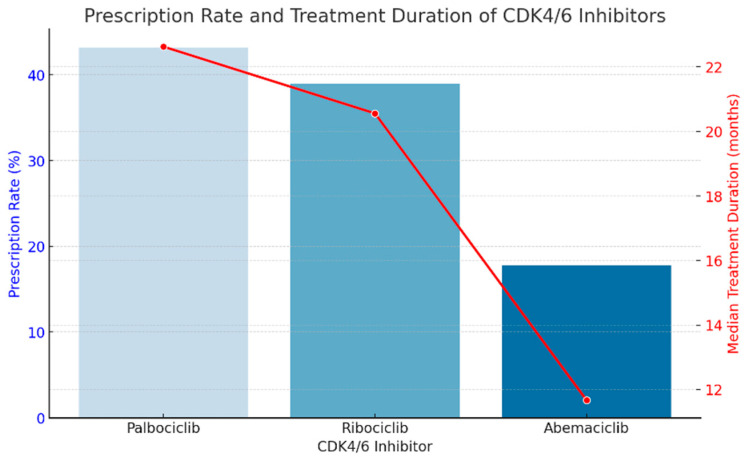
The percentage of prescriptions for each CDK4/6 inhibitor (blue bars) and the median duration of treatment (red line).

We created a combined graph (Figure 1) showing the percentage of prescriptions for each CDK4/6 inhibitor (blue bars) and the median duration of treatment (red line). This graph allows for a clear interpretation of the trends: palbociclib was the most prescribed, and abemaciclib had the shortest duration of treatment, suggesting possible faster disease progression or treatment intolerance.

### 2.3. Toxicity and Dose Modification

Toxicity was reported in 61.1% of the patients, with the most common adverse events being neutropenia (25.5%), thrombocytopenia (16.3%), and gastrointestinal disturbances (14.7%).

The toxicities that we encountered are presented in Table 2. We categorized the toxicities by type and severity according to their Common Terminology Criteria for Adverse Events (CTCAE) grade. Also, one patient reported cutaneous toxicity and one patient reported significant weight loss during CDK4/6i treatment.

Ribociclib and abemaciclib treatments were associated with the highest rates of dose reductions (34.8% and 34.5%, respectively), while palbociclib had a lower rate of dose modifications (23.8%) (Table 3). Neutropenia was the most frequent cause of dose decrease in the ribociclib group, whereas hepatic and digestive toxicity were the most frequent causes for dose reductions of abemaciclib. Neutropenia was the most common condition that the palbociclib group experienced.

Comorbidities such as hypertension, diabetes, and thrombosis were more frequently reported in older patients, with 65.2% of the patients aged over 50 experiencing at least one significant comorbidity. This group also exhibited higher rates of toxicity, likely contributing to dose reductions and adjustments in therapy. The toxicity and survival rates are shown in Table 4.

### 2.4. Survival Outcomes

Overall survival was significantly influenced by patient age and treatment regimen. The 5-year overall survival rate for younger patients was 54.8%, compared to 67.4% for older patients. Ribociclib demonstrated the highest survival benefit, with a 5-year survival rate of 83.3%, followed by abemaciclib (74.7%) and palbociclib (41.5%). Patients treated with palbociclib experienced more frequent treatment interruptions due to toxicity, which may explain the lower survival rates observed in this group.

**Figure 2:**
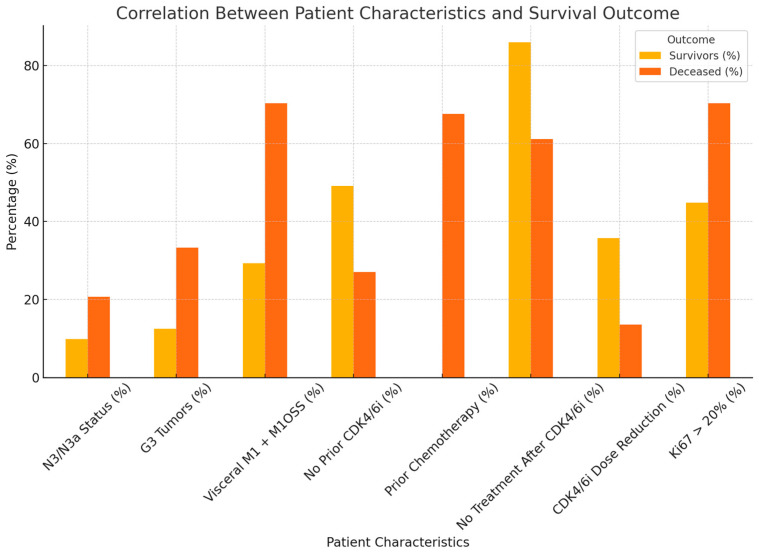
The differences between survivors and deceased patients according to relevant clinical characteristics.

We created a comparative graph (Figure 2) that highlights the differences between the survivors and the deceased patients according to relevant clinical characteristics. It clearly shows that the presence of multiple metastases (Visceral M1 + M1OSS) and a high Ki67 (>20%) were associated with a higher rate of deaths, with statistical significance. Also, reducing the dose of CDK4/6i seems to be associated with better survival.

Next, we wanted to evaluate how the survival outcomes (OS, PFS) and TOT correlated with each CDK4/6i, so we performed a descriptive statistical analysis (Table 5).

**Figure 3:**
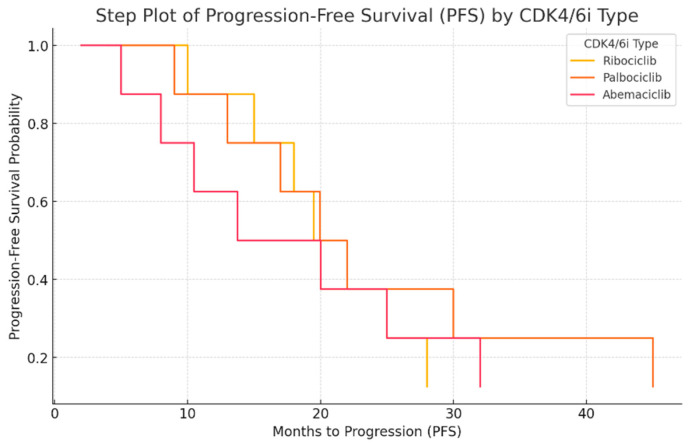
Progression-free survival (PFS) analysis by CDK4/6 inhibitor type.

The next figure presents the Kaplan–Meier-like step plot (Figure 3) for progression-free survival (PFS) by CDK4/6 inhibitor type. This graph represents the probability of remaining progression-free over time. Palbociclib shows the longest PFS, with a slower decline in survival probability. Ribociclib has a moderate PFS, with a slightly steeper drop. Abemaciclib has the shortest PFS, indicating faster disease progression in these patients. This step plot provides a clearer survival probability estimation and is aligned with standard Kaplan–Meier survival analysis.

**Figure 4:**
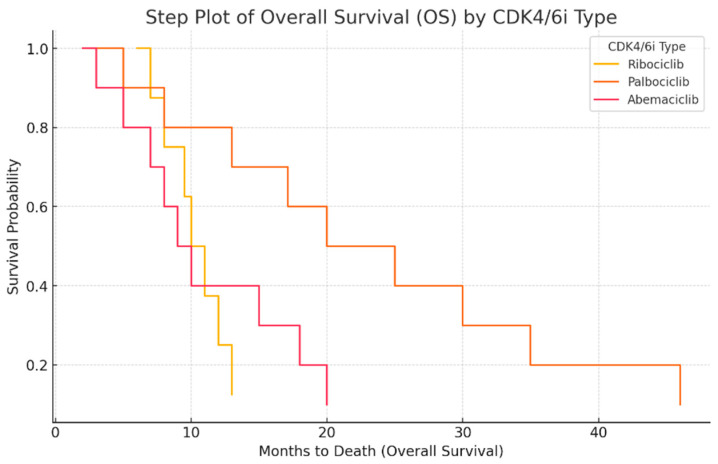
Overall survival (OS) analysis by CDK4/6 inhibitor type.

The next step plot (Figure 4) represents the overall survival (OS) probability for different CDK4/6 inhibitors. This type of graph illustrates how survival probability declines over time. Palbociclib shows the highest survival probability over time, with a slower decline. Ribociclib has a moderate survival curve, showing a more stable decrease. Abemaciclib has the steepest drop, indicating shorter survival duration. This visualization clearly highlights the differences in survival outcomes for different treatments.

Multivariate analysis confirmed that ribociclib treatment, a younger age, and the absence of significant comorbidities were independent predictors of improved overall survival. In contrast, palbociclib was associated with lower survival rates, particularly in older patients with higher comorbidity burdens.

### 2.5. Correlations of Hormonal Therapy and Age with Type of CDK4/6i

The interpretation of the following statistical data focuses on the differences observed according to the type of CDK4/6i, associated hormonal therapy and the age of the patients. We will analyze each combination of variables separately.

The Kaplan–Meier-like step plot for progression-free survival (PFS) shows the probability of being progression-free over time. The Kaplan–Meier-like step plot for overall survival (OS) shows survival probability over time. The box plot for treatment duration (TOT), PFS, and OS shows the distribution and variability of treatment outcomes. The Kaplan–Meier-like plots are ideal for time-to-event analysis, while the box plot is useful for understanding the variability in patient responses.

### 2.6. Differences Observed for Ribociclib

**Figure 5:**
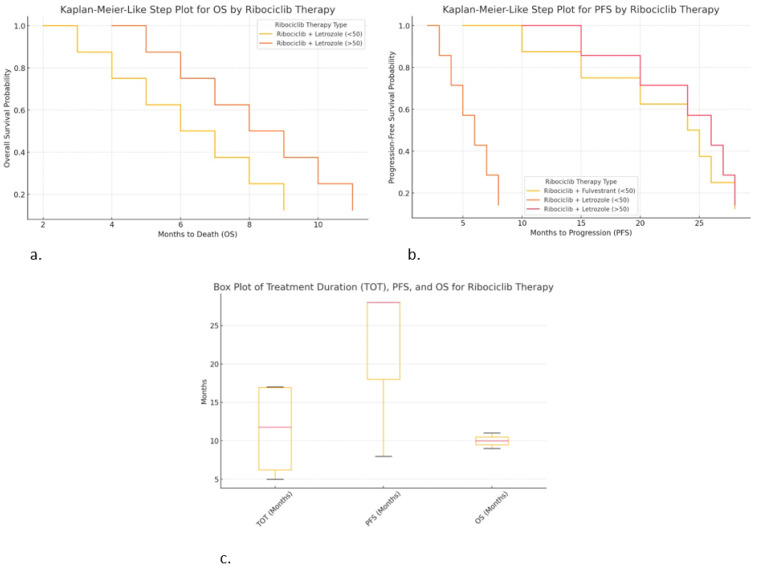
Correlations of hormonal therapy and age with ribociclib: (**a**) Kaplan–Meier-like step plot for PFS; (**b**) Kaplan–Meier-like step plot for OS; (**c**) box plot for TOT, PFS, and OS.

Kaplan–Meier-like step plot for progression-free survival (PFS) (Figure 5a):

○ Ribociclib + Fulvestrant (<50 years) provided longer PFS (up to 28 months).
○ Ribociclib + Letrozole (<50 years) resulted in shorter PFS (~8 months), suggesting earlier progression.
○ Ribociclib + Letrozole (>50 years) resulted in similar PFS (~28 months), suggesting it may work well in older patients.

Kaplan–Meier-like step plot for overall survival (OS) (Figure 5b):

○ Ribociclib + Letrozole (>50 years) resulted in slightly longer OS (up to 11 months).
○ Ribociclib + Letrozole (<50 years) resulted in shorter OS (~9 months).
○ This visualization is useful for comparing the survival outcomes by therapy type.
○ Box plot for TOT, PFS, and OS (Figure 5c):
○ Ribociclib + Fulvestrant (<50 years) resulted in the high consistency in TOT (17 months) and PFS (28 months).
○ Ribociclib + Letrozole (>50 years) shows greater variability in TOT and PFS, suggesting diverse patient responses.
○ Ribociclib + Letrozole (<50 years) resulted in shorter treatment duration and survival, indicating worse outcomes.

### 2.7. Differences Observed for Palbociclib

**Figure 6:**
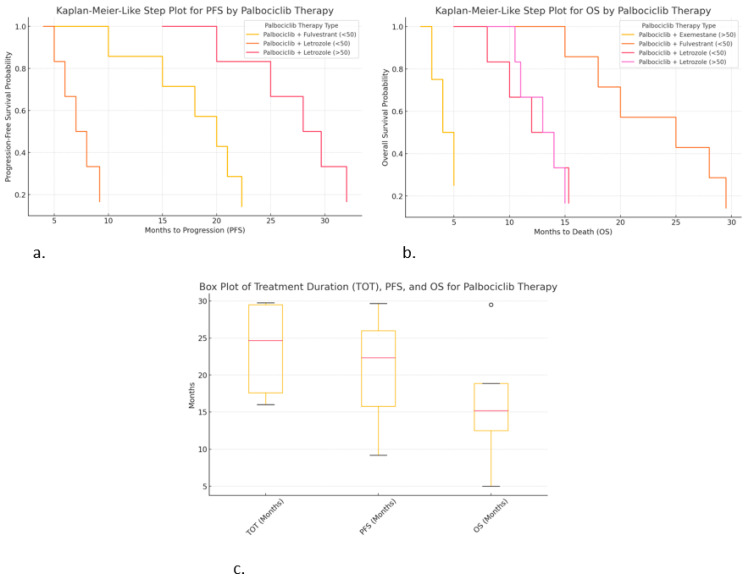
Correlations of hormonal therapy and age with palbociclib: (**a**) Kaplan-Meier-like step plot for PFS; (**b**) Kaplan–Meier-like step plot for OS; (**c**) box plot for TOT, PFS, and OS.

Kaplan–Meier-like step plot for progression-free survival (PFS) (Figure 6a):

○ Palbociclib + Letrozole (>50 years) resulted in the highest PFS (~30 months).
○ Palbociclib + Fulvestrant (<50 years) also shows extended PFS (~22 months).
○ Palbociclib + Letrozole (<50 years) resulted in a much shorter PFS (~9 months), suggesting faster progression.

Kaplan–Meier-like step plot for overall survival (OS) (Figure 6b):

○ Palbociclib + Fulvestrant (<50 years) shows the highest OS (~29.5 months), suggesting significant survival.
○ Palbociclib + Letrozole (>50 years) shows moderate OS (~15 months).
○ Palbociclib + Exemestane (>50 years) resulted in the shortest OS (~5 months), suggesting poor survival in this subgroup.

Box plot for treatment duration (TOT), PFS, and OS (Figure 6c):

○ Palbociclib + Exemestane (>50 years) resulted in high variability in TOT (29.5 months).
○ Palbociclib + Letrozole (>50 years) resulted in the highest PFS (~30 months), with low variability.
○ Palbociclib + Letrozole (<50 years) resulted in lower OS (~15 months), with higher variability.

### 2.8. Differences Observed for Abemaciclib

**Figure 7:**
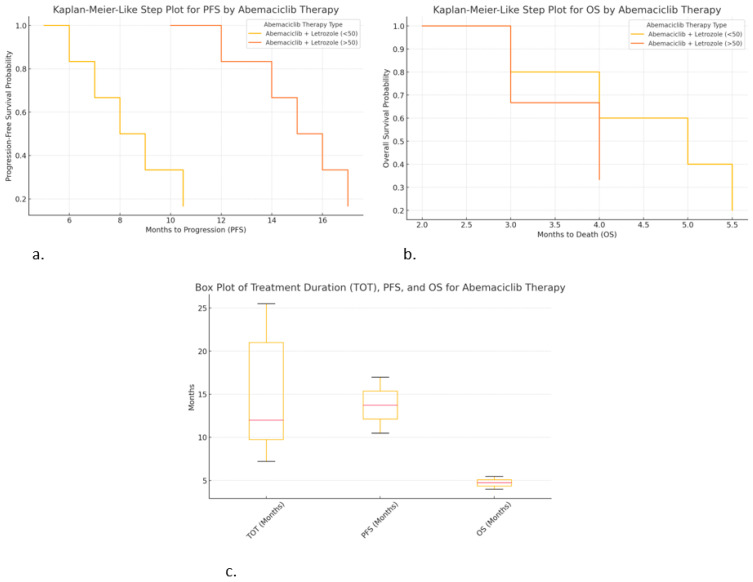
Correlations of hormonal therapy and age with abemaciclib: (**a**) Kaplan–Meier-like step plot for PFS; (**b**) Kaplan–Meier-like step plot for OS; (**c**) box plot for TOT, PFS, and OS.

Kaplan–Meier-like step plot for progression-free survival (PFS) (Figure 7a):

○ Abemaciclib + Letrozole (>50 years) resulted in the longest PFS (~17 months), but it was still lower compared to other CDK4/6i treatments.
○ Abemaciclib + Letrozole (<50 years) resulted in a shorter PFS (~10.5 months), indicating faster progression in younger patients.

Kaplan–Meier-like step plot for overall survival (OS) (Figure 7b):

○ Abemaciclib + Letrozole (<50 years) shows short OS (~5.5 months), indicating poor survival outcomes.
○ Abemaciclib + Letrozole (>50 years) resulted in the worst OS (~4 months, median of ~2 months), suggesting very poor prognosis.

Box plot for treatment duration (TOT), PFS, and OS (Figure 7c):

○ Abemaciclib + Fulvestrant (<50 years) shows the longest TOT (25.5 months).
○ Abemaciclib + Letrozole (>50 years) resulted in high PFS (~17 months) but very low OS (~4 months), suggesting that treatment may delay progression but not improve survival.
○ Variability in TOT was high, especially for Abemaciclib + Letrozole (<50 years), indicating differences in patient responses.

These graphs provide critical insights:

➣ Abemaciclib appears to delay progression but does not significantly improve OS.
➣ Younger patients on abemaciclib may have shorter PFS and OS compared to older patients.
➣ Combination with fulvestrant leads to longer treatment duration (TOT), suggesting better tolerability.

## 3. Materials and Methods

### 3.1. Study Population

This study was conducted on a group of 95 patients diagnosed with HR+/HER2− metastatic breast cancer and treated with CDK4/6 inhibitors. Patient demographics and clinical characteristics were retrospectively extracted from electronic medical records during clinical evaluations at Elias University Emergency Hospital, Bucharest, Romania, from January 2019 to January 2024.

The study protocol was approved by the Ethics Committee of the Elias University Emergency Hospital, Bucharest, Romania. The study design, data analysis, interpretation, drafting, and revisions comply with the Helsinki Declaration and the Committee on Publication Ethics guidelines. All collected data were anonymized, considering the observational nature of the study, without personal data that could lead to the formal identification of the patient (Table 6).

### 3.2. Statistical Analysis

For the statistical processing of the data from the study, IBM SPSS Statistics for Windows software, Version 29.0, was used (30-day trial version) Armonk, NY, USA: IBM Corp. Nominal data were presented as the absolute frequency and percentage, and continuous variables were expressed as the mean, standard deviation, minimum, and maximum. Analysis of the associations among categorical variables was performed using the cross-tabulation and the χ2 (chi-square) test. If the results of the chi-square test were altered enough to not be taken into account, Fisher’s exact test was used. The Mann–Whitney U test was used to compare the means according to the dichotomous variables in the study. The Kruskal–Wallis H test was used to compare the means of the parameters between groups, considering that the variables followed a non-Gaussian distribution. A value of the statistical significance coefficient *p* < 0.05 was considered significant.

Disease progression was defined per RECIST 1.1 criteria, based on radiologic assessment and clinical deterioration. PFS was determined from treatment initiation to radiographic or symptomatic progression.

To control for potential confounding variables, such as prior treatments and comorbidities, in our analysis of progression-free survival (PFS) and overall survival (OS), we employed several statistical methods. First, we conducted multivariable Cox proportional hazards regression analysis, which allowed us to adjust for key factors, such as prior lines of therapy, performance status, comorbidities and other relevant baseline characteristics. This approach enables us to assess the independent effect of CDK4/6 inhibitors on PFS and OS while accounting for these potential confounders.

Additionally, we performed sensitivity analyses, stratifying patients based on factors such as prior treatments and comorbidity burden to evaluate the robustness of our findings. We further included these variables as covariates in the model to ensure that our conclusions were not influenced by their presence.

By implementing these statistical techniques, we aimed to minimize the impact of confounding factors and provide a more accurate estimate of the treatment effect of CDK4/6 inhibitors on PFS and OS in real-world settings.

## 4. Discussion

The present study aimed to analyze the clinical outcomes of treatment with CDK4/6 inhibitors (CDK4/6i) in a cohort of patients with metastatic estrogen receptor (ER)-positive, human epidermal growth factor receptor 2 (HER2)-negative breast cancer, focusing on progression-free survival (PFS), overall survival (OS), and time on treatment (TOT). These data were collected from a real-world setting at a cancer center in Romania, where the impact of CDK4/6i therapy was evaluated across various variables, including treatment type, hormonal therapy combination, age, histological grade, and presence of metastases.

This study revealed that the mean duration of treatment (TOT) with CDK4/6 inhibitors was 16.61 months, with a high degree of variability (standard deviation: 14.353 months), indicating significant heterogeneity in treatment duration across the patient population. This aligns with previous studies that have also reported considerable variation in the time patients remain on CDK4/6i therapy, with some patients receiving treatment for several years, while others discontinue therapy much earlier due to disease progression or toxicity. For instance, in the MONALEESA-7 trial, the median duration of treatment with ribociclib in combination with letrozole was reported to be around 25.3 months, with some patients continuing for longer durations [25].

PFS in our study was found to have a mean of 18.74 months, with a median of 15 months. The variation in PFS (standard deviation: 12.284 months) was considerable, which is in line with results from clinical trials. In the PALOMA-3 trial, the addition of palbociclib to letrozole led to a median PFS of 24.8 months, while in the MONARCH-2 trial, abemaciclib combined with letrozole resulted in a median PFS of 16.4 months [26,27]. These findings support the variation in PFS that we observed, suggesting that while some patients respond favorably to treatment, others experience more rapid progression, particularly with abemaciclib, which had the shortest median PFS in our study (10.50 months).

Similarly, OS in our cohort had a mean of 13.92 months, with a median of 10 months. These results are consistent with several clinical trials that have reported varied OS outcomes with CDK4/6 inhibitors. In the PALOMA-3 trial, patients treated with palbociclib had a median OS of 44.3 months [3]. However, our study observed lower OS values, with palbociclib showing the longest OS (17.13 months) and abemaciclib the shortest (7.33 months). These differences may be attributed to various factors, such as patient characteristics, previous treatments, and comorbidities.

In our analysis, the impact of CDK4/6 inhibitor type on outcomes was also notable. Palbociclib demonstrated the longest mean duration of treatment (22.63 months) and the highest median PFS (19.95 months), supporting its potential efficacy in prolonging disease control. This result is consistent with other studies, such as the PALOMA-3 trial, which demonstrated that palbociclib, when combined with letrozole, significantly prolonged both PFS and OS compared to letrozole alone. Ribociclib had a shorter mean duration of treatment (12.50 months), while abemaciclib had the shortest median duration of treatment (11.67 months). Despite these differences in treatment duration, the differences in PFS and OS among CDK4/6 inhibitors in our study were not statistically significant, suggesting that factors other than the drug type may have a more substantial influence on survival outcomes.

Notably, histological grade, the presence of metastases at diagnosis, and radiotherapy were found to be significantly associated with the duration of treatment. Patients with grade G2 tumors and those with metastatic disease at diagnosis had longer treatment durations (p = 0.045 and p = 0.041, respectively). These results are in line with those of previous studies that suggest poorer prognosis is often associated with higher histological grade and the presence of metastases, which could influence treatment decisions and the intensity of therapy. Furthermore, radiotherapy was associated with shorter treatment duration, a finding which warrants further investigation. The combination of fulvestrant with CDK4/6 inhibitors was associated with longer survival, particularly for patients treated with palbociclib.

An interesting observation from our study was the impact of prior chemotherapy with CDK4/6 inhibitors. Patients who had received prior chemotherapy with CDK4/6i had a longer median OS (21.44 months) compared to those who did not (10.09 months), suggesting a potential benefit of sequencing therapies. This finding highlights the importance of personalized treatment strategies that incorporate previous therapies and the patient’s treatment history, which has been shown to influence both PFS and OS in other real-world studies [28].

Finally, the toxicity profile of CDK4/6 inhibitors also influenced treatment duration. Patients who experienced treatment-related toxicity had a longer duration of therapy (19.29 months) compared to those who did not (12.41 months), which may be reflective of dose adjustments or managed adverse events allowing for prolonged treatment.

## 5. Limitations and Future Directions

While our study offers valuable insights into the real-world efficacy and safety of CDK4/6 inhibitors, it is not without limitations. The sample size was relatively small, and the cohort was heterogeneous in terms of age, prior treatments, and comorbidities, which could influence the applicability of the findings. Despite these challenges, our results provide valuable real-world insights that complement existing randomized clinical trial data. However, prospective studies are needed to further validate our findings in a controlled setting. Future research should also explore the genetic and molecular determinants that influence patient response to CDK4/6 inhibitors, as well as the optimal sequencing of therapies in metastatic ER-positive breast cancer.

We recognize the clinical relevance of discussing the effects of CDK4/6 inhibitors in the context of molecular subtypes of HR+/HER2-negative/non-luminal breast cancer, as it was presented in two major analyses by Cejalvo et al. in 2017 [29] and 2018 [30]. However, a detailed analysis of this aspect extends beyond the scope of the present study, and we plan to address this important topic in a future study.

## 6. Conclusions

This study provides new insights into the differential efficacy of CDK4/6 inhibitors in metastatic and locally advanced breast cancer, emphasizing the importance of personalized treatment strategies. Younger patients, despite receiving similar treatments, exhibited poorer survival outcomes, likely due to higher tumor aggression (as evidenced by increased Ki67 and higher histological grade) and more advanced disease at diagnosis.

Ribociclib showed the greatest efficacy, particularly in patients with lower comorbidity burdens, while palbociclib treatment was associated with a higher rate of toxicity and dose reductions. These findings underscore the need for careful management of older patients, who often present with more comorbidities and may require dose adjustments to optimize survival outcomes.

These findings advocate for personalized treatment approaches, considering both patient-specific and tumor-related factors to optimize clinical outcomes.

## Figures and Tables

**Table 1:** Patient demographic and clinical characteristics.

Parameters	CDK		
	Ribociclib	Palbociclib	Abemaciclib
Environment—*n* (%)			
Rural	1 (4.2%)	8 (19.5%)	5 (16.7%)
Urban	23 (95.8%)	33 (80.5%)	25 (83.3%)
Age—mean (±SD)	53.67 (±15.11)	57.68 (±13.35)	59.00 (±10.47)
Lymph node status at diagnosis			
N0	5 (21.7%)	6 (20.0%)	8 (29.6%)
N1	8 (34.8%)	8 (26.7%)	12 (44.4%)
N1a	2 (8.7%)	3 (10.0%)	1 (3.7%)
N2	3 (13.0%)	5 (16.7%)	3 (11.1%)
N2a	2 (8.7%)	2 (6.7%)	1 (3.7%)
N3	2 (8.7%)	5 (16.7%)	1 (3.7%)
N3a	1 (4.3%)	1 (3.3%)	1 (3.7%)
Histological grade at diagnosis			
G1	0 (0.0%)	5 (13.5%)	3 (10.3%)
G2	23 (100.0%)	24 (64.9%)	16 (55.2%)
G3	0 (0.0%)	8 (21.6%)	10 (34.5)
Non-metastatic/metastatic at diagnosis			
Non-metastatic	14 (58.3%)	24 (58.5%)	18 (60.0%)
Metastatic	10 (41.7%)	17 (41.5%)	12 (40.0%)
Metastasis			
without	0 (0.0%)	0 (0.0%)	6 (20.0%)
Visceral M1, without bone, lymphatic, or skin lesions	4 (16.7%)	7 (17.1%)	6 (20.0%)
M1OSS	7 (29.2%)	12 (29.3%)	7 (23.3%)
Visceral Mi + M1OSS	12 (50.0%)	21 (51.2%)	10 (33.3%)
Other	1 (4.2%)	1 (2.4%)	1 (3.3%)
Treatment prior to CDK4/6i therapy			
Without chemotherapy	11 (45.8%)	14 (34.1%)	13 (44.8%)
With chemotherapy	11 (45.8%)	26 (63.4%)	13 (44.8%)
With hormonotherapy	2 (8.3%)	1 (,4%)	3 (10.3%)
Treatment administered after CDK4/6i therapy			
Without chemotherapy	20 (83.3%)	23 (59.0%)	28 (93.3%)
With chemotherapy	2 (8.3%)	12 (30.8%)	2 (6.7%)
With hormonotherapy and chemotherapy	2 (8.3%)	4 (10.3%)	0 (0.0%)
Toxicity of CDK4/6i			
Without toxicity	9 (37.5%)	15 (36.6%)	13 (43.3%)
With toxicity	15 (62.5%)	26 (63.4%)	17 (56.7%)
Reduction of CDK4/6i dose			
No	15 (65.2%)	34 (82.9%)	19 (65.5%)
Yes	8 (34.8%)	7 (17.1%)	10 (34.5%)
Type of hormone therapy used in combination with CDK4/1			
Anastrozole	0 (0.0%)	0 (0.0%)	1 (3.3%)
Exemestane	0 (0.0%)	2 (4.9%)	1 (3.3%)
Fulvestrant	5 (20.8%)	9 (22.0%)	5 (16.7%)
Letrozole	19 (79.2%)	30 (73.2%)	23 (76.7%)
Menopausal status at initiation of CDK4/6i treatment			
Premenopause	7 (29.2%)	6 (14.6%)	4 (13.3%)
Menopause	17 (70.8%)	35 (85.4%)	26 (85.4%)
Ki67 percentage			
Below 20%	13 (54.2%)	15 (36.6%)	15 (50.0%)
Above 20%	11 (45.8%)	26 (63.4%)	15 (50.0%)
Patient has thrombosis			
No	19 (79.2%)	32 (78.0%)	21 (75.0%)
Yes	5 (20.8%)	9 (22.0%)	7 (25.0%)
Associated comorbidities			
No	4 (16.7%)	6 (14.6%)	6 (20.0%)
Yes	20 (83.3%)	35 (85.4%)	24 (80.0%)
Smoking			
No	21 (87.5%)	39 (95.1%)	25 (83.3%)
Yes	3 (12.5%)	2 (4.9%)	5 (16.7%)
Oncological family history			
No	14 (58.3%)	33 (80.5%)	23 (76.7%)
Yes	10 (41.7%)	8 (19.5%)	7 (23.3%)
Death/survival at time of study			
Survival	20 (83.3%)	17 (41.5%)	21 (70.0%)
Death	4 (16.7%)	24 (58.5%)	9 (30.0%)
Patient underwent mastectomy			
No	9 (37.5%)	23 (56.1%)	14 (46.7%)
Yes	15 (62.5%)	18 (43.9%)	16 (53.3%)
Patient underwent radiotherapy			
No	9 (37.5%)	15 (36.6%)	11 (37.9%)
Yes, at breast level	8 (33.3%)	10 (24.4%)	8 (27.6%)
Yes, at other level	5 (20.8%)	12 (29.3%)	6 (20.7%)
At breast level + other levels	2 (8.3%)	4 (9.8%)	4 (13.8%)

**Table 2:** Toxicities to CDK4/6 inhibitors during treatment. N = number of patients.

Toxicity	Grade 1 (N)	Grade 2 (N)	Grade 3 (N)	Grade 4 (N)
Neutropenia	1	0	26	7
Thrombocytopenia	5	5	7	9
Digestive toxicity	2	2	6	0
Hepatic toxicity	1	6	4	1
Anemia	0	0	2	3
Hyperbilirubinemia	0	0	3	0
Renal toxicity	0	2	0	0

**Table 3:** Toxicity, dose reduction, and associated comorbidities.

Parameters	N (%)	Age	
		<50 Years	>50 Years
Toxicity to CDK4/6i			
Without toxicity	37 (38.9%)	13 (41.9%)	24 (37.5%)
With toxicity	58 (61.1%)	18 (58.1%)	40 (62.5%)
CDK4/6i dose reduction			
No	68(73.1%)	24 (77.4%)	44 (71.0%)
Yes	25 (26.9%)	7 (22.6%)	18 (29.0%)
Patient has thrombosis			
No	72 (77.4%)	25 (80.6%)	47 (75.8%)
Yes	21 (22.6%)	6 (19.4%)	15 (24.2%)
Associated comorbidities			
No	16 (16.8%)	6 (19.4%)	10 (15.6%)
Yes	79 (83.2%)	25 (80.6%)	54 (84.4%)

**Table 4:** Toxicity and survival rates.

Toxicity to CDK4/6i		N	Mean	Standard Deviation
Number of months of CDK4/6i treatment (TOT)	No toxicity	37	12.41	11.800
	With toxicity	58	19.29	15.264
	Total	95	16.61	14.353
Number of months to progression (PFS)	No toxicity	11	16.55	10.103
	With toxicity	16	20.25	13.694
	Total	27	18.74	12.284
Number of months to death (OS)	No toxicity	16	13.00	10.621
	With toxicity	21	14.62	13.079
	Total	37	13.92	11.945

**Table 5:** Detailed interpretation of statistical data regarding the evaluation of PFS, OS, and TOT according to the type of CDK4/6i.

	CDK4/6i	Mean	Median	Domi-Nance	Standard Deviation	Min	Max
No. of months of treatment with CDK4/6i (TOT)	Ribociclib	12.50	8.00	5	9.673	2	33
	Palbociclib	22.63	20.00	5	16.803	2	63
	Abemaciclib	11.67	7.00	2 ^a^	10.656	2	36
No. of months to progression (PFS)	Ribociclib	18.00	19.50	28	11.804	5	28
	Palbociclib	19.95	17.00	13 ^a^	12.567	4	45
	Abemaciclib	13.75	10.50	2 ^a^	13.326	2	32
No. of months until death (OS)	Ribociclib	9.50	9.50	6 ^a^	3.109	6	13
	Palbociclib	17.13	13.00	5	13.349	2	46
	Abemaciclib	7.33	7.00	2	6.062	2	20

^a^ Multiple modes exist. The smallest value is shown.

**Table 6:** Inclusion and exclusion criteria.

Inclusion Criteria	Exclusion Criteria
Female patients aged ≥ 18 years. Either treated or currently in treatment with CDK4/6 inhibitors, with pathologically confirmed HR+/HER2− breast cancer.	Male patients or females aged < 18 years. Patients with <3 months of CDK4/6 inhibitor therapy.
Minimum of three presentations in our oncology department.	

Disease progression was verified by the individual clinicians at the oncology department and included clinical, radiological, and biochemical measures.

## Data Availability

The data are available upon request from the first author, due to privacy and ethical restrictions.
